# Genome Sequence of *Paenibacillus polymyxa* Strain CICC 10580, Isolated from the Fruit of Noni (*Morinda citrifolia* L.) Grown in the Paracel Islands

**DOI:** 10.1128/genomeA.00854-14

**Published:** 2014-08-28

**Authors:** Youqiang Xu, Yang Liu, Su Yao, Jinxia Li, Chi Cheng

**Affiliations:** a China National Research Institute of Food and Fermentation Industries, Beijing, China; b China Center of Industrial Culture Collection, Beijing, China

## Abstract

Noni is a plant reported to have nutritional and therapeutic properties. *Paenibacillus polymyxa* CICC 10580 is a strain that was isolated from the fruit of noni and showed comprehensive antagonistic activity against many pathogens. Its genome was sequenced and assembled (6.10 Mb). The coding sequences (CDSs) correlated with antagonistic activity were annotated.

## GENOME ANNOUNCEMENT

*Morinda citrifolia* L., commonly known as noni, is a tropical and subtropical plant that has been used traditionally as a folk medicine to treat a wide range of diseases for thousands of years (1, 2). Noni products have been reported to have health-related benefits such as analgesic, anti-inflammatory, and cardiovascular activities and anticancer and antioxidant properties (3–5). Researchers have isolated a series of biological compounds such as polysaccharides, glycosides, iridoids, lignans, anthraquinones, trisaccharide fatty acid esters, scopoletin, and vitamins from the noni plant (5–11). However, few studies were concerned with the endophytic bacteria that play important roles in the growth and functional chemical synthesis of plants (12). The strain *Paenibacillus polymyxa* CICC 10580 was isolated from the fruit of noni that grew in the Paracel islands.

Here we report the draft genome sequence of *Paenibacillus polymyxa* CICC 10580, which was determined by Illumina HiSeq 2000 (13). The genome sequence contained 5,108,612 reads for shotgun sequencing and 6,625,600 reads for paired-end sequencing. The reads were assembled using the SOAPdenovo 2.04 system (14, 15) into 23 contigs (>200 bp) with a length of 6,099,109 bp and a G+C content of 45.3%. The genome sequence was annotated by the RAST server (16). tRNAs and rRNAs were predicted by tRNAscan-SE v. 1.23 (17) and RNAmmer 1.2 (18), respectively.

The genome sequence of strain CICC 10580 contained 5,375 protein-coding sequences (CDSs). A total of 124 tRNAs and 41 rRNAs were identified. Three CDSs for glucanase, 4 CDSs for cellulase, and 2 CDSs for chitinase were annotated and correlated to antagonistic activity (19–21), which was in accordance with experimental results (unpublished data). In addition, strain CICC 10580 contained 31, 18, and 7 CDSs for synthesis of the vitamins biotin, thiamin, and pyridoxine, respectively. The strain also has 24 CDSs for folate synthesis, 16 CDSs for exopolysaccharide synthesis, and 21 CDSs for sialic acid synthesis. These annotated CDSs indicate that strain CICC 10580 may play an important role in functional chemical synthesis in the noni plant. The genome sequence and annotation of *Paenibacillus polymyxa* CICC 10580 may promote further studies of the interrelationships between the endophytic bacteria and the noni plant as well as its antagonistic activity and therapeutic and nutritional properties.

### Nucleotide sequence accession number.

This whole-genome shotgun project has been deposited at DDBJ/EMBL/GenBank under the accession number JNCB00000000. The version described in this paper is version JNCB01000000.
